# RGD-modified multifunctional nanoparticles encapsulating salvianolic acid A for targeted treatment of choroidal neovascularization

**DOI:** 10.1186/s12951-021-00939-9

**Published:** 2021-07-02

**Authors:** Junxiu Zhang, Jingyi Zhu, Lingzhou Zhao, Ke Mao, Qing Gu, Dongli Li, Jinhua Zhao, Xingwei Wu

**Affiliations:** 1 Department of Ophthalmology, Shanghai General Hospital, Shanghai Jiao Tong University School of Medicine, Shanghai Key Laboratory of Ocular Fundus Diseases, Shanghai Engineering Center for Visual Science and Photomedicine, Shanghai, 200080 People’s Republic of China; 2 School of Pharmaceutical Sciences, Nanjing Tech University, Nanjing, 211816 People’s Republic of China; 3 Department of Nuclear Medicine, Shanghai General Hospital, Shanghai Jiao Tong University School of Medicine, 200080 Shanghai, People’s Republic of China; 4 Department of Ophthalmology, Renji Hospital, School of Medicine, Shanghai Jiao Tong University, Shanghai, 200127 People’s Republic of China

**Keywords:** Choroidal neovascularization, Salvianolic acid A, Polyethyleneimine, Anti-angiogenesis, SPECT imaging

## Abstract

**Background:**

The development of alternative anti-angiogenesis therapy for choroidal neovascularization (CNV) remains a great challenge. Nanoparticle systems have emerged as a new form of drug delivery in ocular diseases. Here, we report the construction and characterization of arginine-glycine-aspartic acid (RGD)-conjugated polyethyleneimine (PEI) as a vehicle to load antioxidant salvianolic acid A (SAA) for targeted anti-angiogenesis therapy of CNV. In this study, PEI was consecutively modified with polyethylene glycol (PEG) conjugated RGD segments, 3-(4′-hydroxyphenyl) propionic acid-Osu (HPAO), and fluorescein isothiocyanate (FI), followed by acetylation of the remaining PEI surface amines to generate the multifunctional PEI vehicle PEI.NHAc-FI-HPAO-(PEG-RGD) (for short, RGD-PEI). The formed RGD-PEI was utilized as an effective vehicle platform to load SAA.

**Results:**

We showed that RGD-PEI/SAA complexes displayed desirable water dispersibility, low cytotoxicity, and sustainable release of SAA under different pH conditions. It could be specifically taken up by retinal pigment epithelium (RPE) cells which highly expressed ɑ_v_β_5_ integrin receptors in vitro and selectively accumulated in CNV lesions in vivo. Moreover, the complexes displayed specific therapeutic efficacy in a mouse model of laser induced CNV, and the slow elimination of the complexes in the vitreous cavity was verified by SPECT imaging after ^131^I radiolabeling. The histological examinations further confirmed the biocompatibility of RGD-PEI/SAA.

**Conclusions:**

The results suggest that the designed RGD-PEI/SAA complexes may be a potential alternative anti-angiogenesis therapy for posterior ocular neovascular diseases.

**Graphic abstract:**

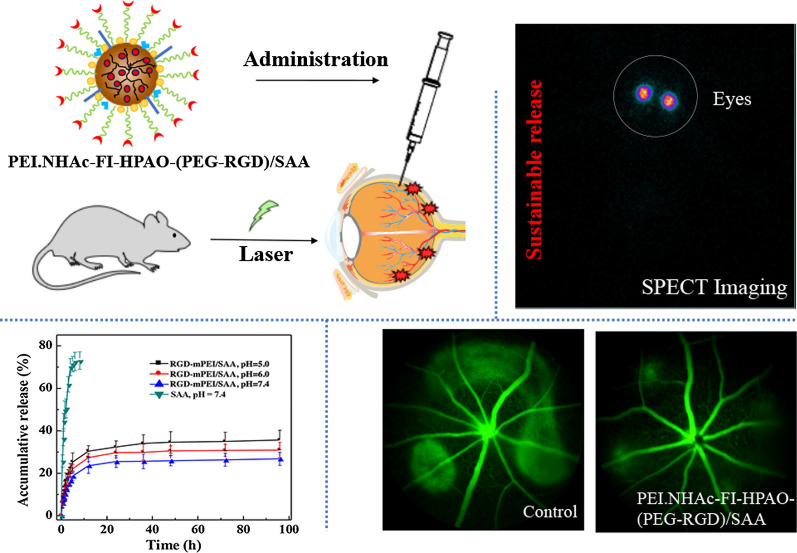

**Supplementary Information:**

The online version contains supplementary material available at 10.1186/s12951-021-00939-9.

## Introduction

Age-related macular degeneration (AMD) is the leading cause of blindness and visual loss among elderly patients [1, 2]. The number of AMD patients in the world is estimated to be 196 million in 2020 and 280 million in 2040 [3]. This disease can be classified as non-exudative geographic atrophy (‘‘dry’’ AMD) and exudative neovascularization (‘‘wet’’ AMD). Although wet AMD is experienced by only 10–15% of patients, it is responsible for almost all of the rapid vision loss of the entire AMD population [4, 5]. The pathological characterization of wet AMD is choroidal neovascularization (CNV), the occurrence of neovascular vessels originating from the choriocapillaris, which can subsequently lead to vessel leakage in the sub-retinal space and sudden vision loss [6].

Currently, monthly intravitreal injection of antibodies against vascular endothelial growth factor (VEGF) is considered the best therapy for AMD [7–9]. However, in the clinic, more than 60% of AMD patients are resistant to the current therapy and suffer from serious side effects, including endophthalmitis, retinal detachment, and laser-inducing destruction of normal retina [10]. As VEGF also plays a vital role in retinal tissue, VEGF deprivation therapy might be the reason for original geographic atrophy in patients with wet AMD [11, 12]. In addition, oxidative damage is widely believed to play a central role during angiogenesis [13, 14]. Oxidized products such as hydrogen peroxide could improve VEGF expression in endothelial cells and vascular smooth muscle cells, inducing angiogenic responses [15–17]. Oxidized low-density lipoproteins (ox-LDL) generated by oxidative stress could also increase VEGF expression in human macrophages and tube formation in endothelial cells [18, 19]. Thus, the inhibition of both VEGF and oxidative pathways may produce a synergistic effect on angiogenesis. What’s more, the development of targeting angiogenesis agents with sustained-release properties remains a big clinical challenge [20].

Salvianolic acids are extracted from *Salvia miltiorrhiza* Bunge (Danshen), which has a long history of use in the treatment of liver and heart diseases in China [21, 22]. Salvianolic acid A (SAA), the strongest antioxidant among salvianolic acids, is a potent free radical scavenger due to its polyphenolic structure [23]. Some researchers have reported that SAA can protect the myocardium by reducing oxidative stress [24–26]. In our previous work, we revealed that ox-LDL could induce chronic oxidative damage and inflammation to the RPE layer and exacerbate CNV progression, which could be reversed by SAA [27, 28]. Therefore, SAA could be a promising therapeutic agent for wet AMD [29]. However, several shortcomings of salvianolic acid have been demonstrated, such as short vitreal half-life, low bioavailability, poor stability, and rapid decomposition in aqueous media, which limit its clinical use [30, 31].

Nanotechnology offers great advantages for overcoming these limitations due to its unique properties. Among nanotechnology applications, polyethyleneimine (PEI) is a class of cationic polymers with abundant positive surface charge and shows potential for use as a drug delivery vector [32, 33]. Arginine-glycine-aspartate (RGD) peptide is a common strategy for nanoparticles to target neovascular lesions by binding to integrins [34, 35]. In terms of physical conditions, the RPE requires integrin ɑ_v_β_5_ to recognize spent photoreceptor outer segment particles, which is critical for vision [36–38]. In ocular angiogenesis, integrin ɑ_v_β_3_ is reported to be overexpressed on the CNV membrane [39–41]. The previous success of RGD-modified PEI-based multifunctional nanoparticles [42] inspired us to speculate that RGD-modified PEIs could also be used as a nanoplatform to load SAA and might be able to increase the aqueous solubility of SAA, prevent its degradation, and target RPE cells and angiogenesis.

The current work aimed to build an RGD-modified PEI-entrapped SAA nanosystem for the targeted treatment of CNV. We prepared and characterized multifunctional PEI.NHAc-FI-HPAO-(PEG-RGD)/SAA (for short, RGD-PEI/SAA). A cell counting kit-8 (CCK-8) assay was used to assess the cytotoxicity of the nanoparticles. The targeting specificity of the RGD-PEI/SAA complex was evaluated by confocal laser scanning microscopy (CLSM), flow cytometry in human retinal pigment epithelium cell line (ARPE-19) cells and a laser-induced CNV mouse model in vivo. Hematoxylin and eosin (HE) staining, fundus fluorescein angiography (FFA) imaging and grading, and choroid flat-mount were used to evaluate the therapeutic efficiency in laser-induced CNV mice models. Tube formation assay and wound healing assay were used to evaluate the anti-vascular effect in vitro. Single-photon emission computerized tomography (SPECT) imaging was used to study the pharmacokinetics after intravitreal injection. As far as we know, this is the first study of RGD-modified PEI polymer encapsulating SAA for targeting angiogenesis therapy in laser-induced CNV mice models.

## Experimental section

### Synthesis of ^131^I-RGD-PEI/SAA

RGD-PEI/SAA complexes were prepared according to a previously reported protocol [32, 33]. In brief, 40 mg RGD-PEG-COOH dissolved in DMSO were activated by EDC and was then mixed with PEI.NH_2_ (300 mg) in DMSO solution under constant stirring at room temperature for 3 days to obtain PEI.NH_2_-(PEG-RGD). The mixture was then added to HPAO (31.6 mg) and FI (7.8 mg) with stirring overnight to obtain PEI.NH_2_-FI-HPAO-(PEG-RGD). The remaining PEI surface amines were acetylated by excess Ac_2_O, and PEI.NHAc-FI-HPAO-(PEG-RGD) (for short, RGD-PEI) was obtained. After purification following the protocols published in our previous work to remove the excess reactants and byproducts, the obtained RGD-PEI was used as a template to load SAA. Here, SAA was encapsulated into the RGD-PEI nanoplatform through electrostatic interaction. SAA (20 mg) was dissolved in methanol and added to an aqueous RGD-PEI solution (125.1 mg in 5 mL water), and the mixture was vigorously stirred overnight to evaporate the methanol solvent. The mixture was then centrifuged (8000 rpm) for 10 min to remove the precipitate and collect the supernatant solution. After lyophilization, RGD-PEI/SAA complexes were obtained. For comparison, PEI.NHAc-FI-HPAO-*m*PEG/SAA (for short, *m*PEI/SAA) without RGD modification was also synthesized. Finally, ^131^I was labeled with the HPAO segments of RGD-PEI/SAA using the chloramine T method. Two hundred *µ*L of PBS solution containing 200 *µ*g RGD-PEI/SAA and 200 *µ*g chloramine T were mixed with 100 *µ*L of Na^131^I solution (10 mCi). After incubation for 30 min at 37 °C under continuous stirring, the reaction mixture was purified through PD-10 desalting columns (cut-off value 5000 Da) using PBS as the mobile phase to separate free ^131^I and ^131^I-RGD-PEI/SAA (Mw = 62721.85). The purified ^131^I-RGD-PEI/SAA was collected in a tube according to our previous work. According to a similar labeling strategy, ^131^I-PEI/SAA and ^131^I-SAA were also prepared.

### Characterization techniques

^1^H nuclear magnetic resonance (^1^H NMR) spectra of the intermediate products dissolved in D_2_O, including PEI.NH_2_-(PEG-RGD), PEI.NH_2_-HPAO-(PEG-RGD), PEI.NH_2_-FI-HPAO-(PEG-RGD), PEI.NH_2_-(*m*PEG), PEI.NH_2_-HPAO-(*m*PEG) and PEI.NH_2_-FI-HPAO-(*m*PEG), were obtained using a Bruker AV400 nuclear magnetic resonance spectrometer (Bruker AXS Advanced X-ray Solutions GmbH, Karlsruhe, Germany). UV–vis spectra were measured using a Lambda 25 UV–vis spectrophotometer (PerkinElmer, Inc., Waltham, MA, USA). Dynamic light scattering (DLS) and zeta potential measurements were performed using a Malvern Zetasizer Nano ZS model ZEN 3600 (Malvern Panalytical Ltd., Malvern, UK) with a standard 633 nm laser. Instant thin-layer chromatography (ITLC) was used to assess the radiochemical yields (RCYs) and stabilities of the ^131^I labeled complexes and ^131^I-SAA. ITLC was performed using silica gel-coated fiber glass sheets (Macherey-Nagel, GmbH & Co. KG, Düren, Germany) using PBS as the mobile phase. The sheets were analyzed using a thin layer chromatogram scanner (Bioscan Inc., Tucson, AZ).

### In vitro drug release

The RGD-PEI/SAA complexes (3 mg) were dispersed in 1 mL of PBS (pH 7.4) or acetate buffer (pH 5.0 and 6.0) and placed in a dialysis bag (MWCO = 14,000) for release kinetic studies [42, 43]. The dialysis bag was then immersed in the corresponding buffer medium with a volume of 9 mL and incubated at 37 °C. At different time points, 1 mL of the outer phase medium was removed and quantified by UV-vis spectroscopy, and the same volume of fresh corresponding buffer medium was replenished. As a control, the release experiment of free SAA was carried out using the same method at a pH of 7.4.

### Cytotoxicity assay

The CCK-8 assay was used to assess the cytotoxicity of RGD-PEI, RGD-PEI/SAA, PEI.NHAc-FI-HPAO-(*m*PEG) (*m*PEI), and free SAA in ARPE-19 cells [42, 43]. ARPE-19 cells were seeded on a coverslip of a 24-well plate to evaluate the effect of the materials on the cytoskeleton. After 24 h of starvation, the cells were incubated with RGD-PEI/SAA at SAA concentrations of 0, 20, 50, and 100 *µ*M for 24 h. Then, the images of the cytoskeleton were obtained under fluorescence microscope.

### Flow cytometry assay of cellular uptake

Flow cytometry was used to evaluate the targeting specificity of the RGD-PEI/SAA or nontargeted PEI/SAA complexes to ARPE-19 cells. After 24 h of starvation, the cells were incubated with RGD-PEI/SAA or PEI/SAA nanoparticles at a 20 *µ*M concentration of SAA for 4 h. After trypsinization, centrifugation, and suspension in 1 mL of PBS, the cells were finally analyzed following a previously described protocol [43].

### Confocal laser scanning microscopy (CLSM)

Confocal microscopic analysis using a Carl Zeiss LSM 700 laser scanning confocal microscope (Jena, Germany) was performed to evaluate the targeting specificity of the RGD-PEI/SAA under an SAA concentration of 20 *µ*M [43].

### Tube formation assay

Tube formation assays were carried out following previous protocols [44]. In brief, 96-well plates were coated with Matrigel and human umbilical vein endothelial cells (HUVECs) were seeded on Matrigel matrix. SAA (20 *µ*M), RGD-PEI/SAA, and *m*PEI/SAA at equal SAA concentrations, or the corresponding concentrations of RGD-PEI and *m*PEI were added immediately. The cells without any treatment were used as controls. After 8 h of incubation, the endothelial tube formation was observed using a fluorescence microscope and the tube-like structures were counted.

### Wound healing assay

The in vitro HUVEC scratch assay was performed as described previously [44]. In brief, HUVECs were seeded and incubated overnight. The next day, two separate straight-line scratches were created with a 1 mL pipette in the middle of the monolayer. The media were then changed with or without SAA, RGD-PEI/SAA, and *m*PEI/SAA at 20 *µ*M SAA or equal concentrations of RGD-PEI and *m*PEI. The images were acquired at 0 and 24 h using an optical microscope. The experiment was repeated in triplicate.

### SPECT imaging

The synthesized ^131^I-RGD-PEI/SAA, ^131^I-PEI/SAA, ^131^I-SAA, and Na^131^I were intravitreally injected into mice after laser burn, respectively. Then, these mice were scanned by a SPECT imaging system using a GE Infinia SPECT scanner equipped with an Xeleris workstation and high-energy general-purpose collimators (GE Healthcare). SPECT images were acquired at 6, 24, 48, 72, and 96 h after laser injury and intravitreal injection, respectively.

### Animals

Healthy C57BL/6J mice (6–8 weeks old) weighing 20–30 g were obtained from the Shanghai Laboratory Animal Center of the Chinese Academy of Sciences. All the experiments involving animals were approved by the ethical committee of Shanghai General Hospital and performed in accordance with Association for Research in Vision and Ophthalmology (ARVO) statements for the use of animals in ophthalmic and vision research.

### Laser-induced choroidal neovascularization (CNV)

CNV was carried out following previous protocols [44]. In brief, 4–5 spots of laser photocoagulation were placed in each eye using the Micron IV retinal image-guided laser system (Phoenix Research Laboratories). The appearance of cavitation bubbles confirmed the success of Bruch’s membrane disruption. Intravitreal injections were performed after photocoagulation. A 35-gauge needle attached to a 10 *µ*L nanofil syringe (World Precision Instruments, Sarasota, FL, USA) was used to deliver 2 *µ*L of SAA dissolved in PBS (20 *µ*M) into the vitreous cavity, and PBS was used as a control. Mice were kept in a temperature-controlled room under a 12-hour dark/light circle for another 7 days before the endpoint of our study.

### Fundus fluorescein angiography (FFA) imaging

The formation of CNV lesions was monitored and evaluated after intraperitoneal injection of 100 *µ*L of 10% fluorescein sodium using a Micron IV imaging system.

### Quantification of CNV

Fundus images of the mice were taken 7 days after the laser injury. Choroidal flat mounts of the eyes were prepared according to previous protocols [27, 28]. After fixation for 4 h in paraformaldehyde, the muscles, anterior segments, and the lens of the fixed eyes were carefully removed. The neural retinas of the eyecups were gently seperated and the remaining RPE/choroid/sclera complexes were made into 4–5 radial incisions towards the optic nerve head using fine-curved scissors. After washing in cold PBS buffer, the RPE/choroid/sclera complexes were incubated in isolectin B4 (Invitrogen, Cat. No. 121,411) at a dilution of 1:200 overnight at 4 °C. Each flat mount was finally washed with 1× PBS, mounted with antifade mounting media (VECTASHIELD; Vector Laboratories, CA), covered, and sealed before observation using a microscope (Carl Zeiss, Norway). The fluorescence images were analyzed using Image J software following an earlier method [44, 45].

### Hematoxylin and eosin (HE) staining

A histopathological study was performed as previously described [27, 46]. Briefly, enucleated eyes were fixed for 24 h. After embedding in paraffin, sectioning, and staining with hematoxylin and eosin, images of CNV were observed under fluorescence microscope and measured using Image J software by a pathologist in a blind study.

## Results and discussion

### Synthesis and characterization of ^131^I-PEI-RGD/SAA complexes

**Fig. 1:**
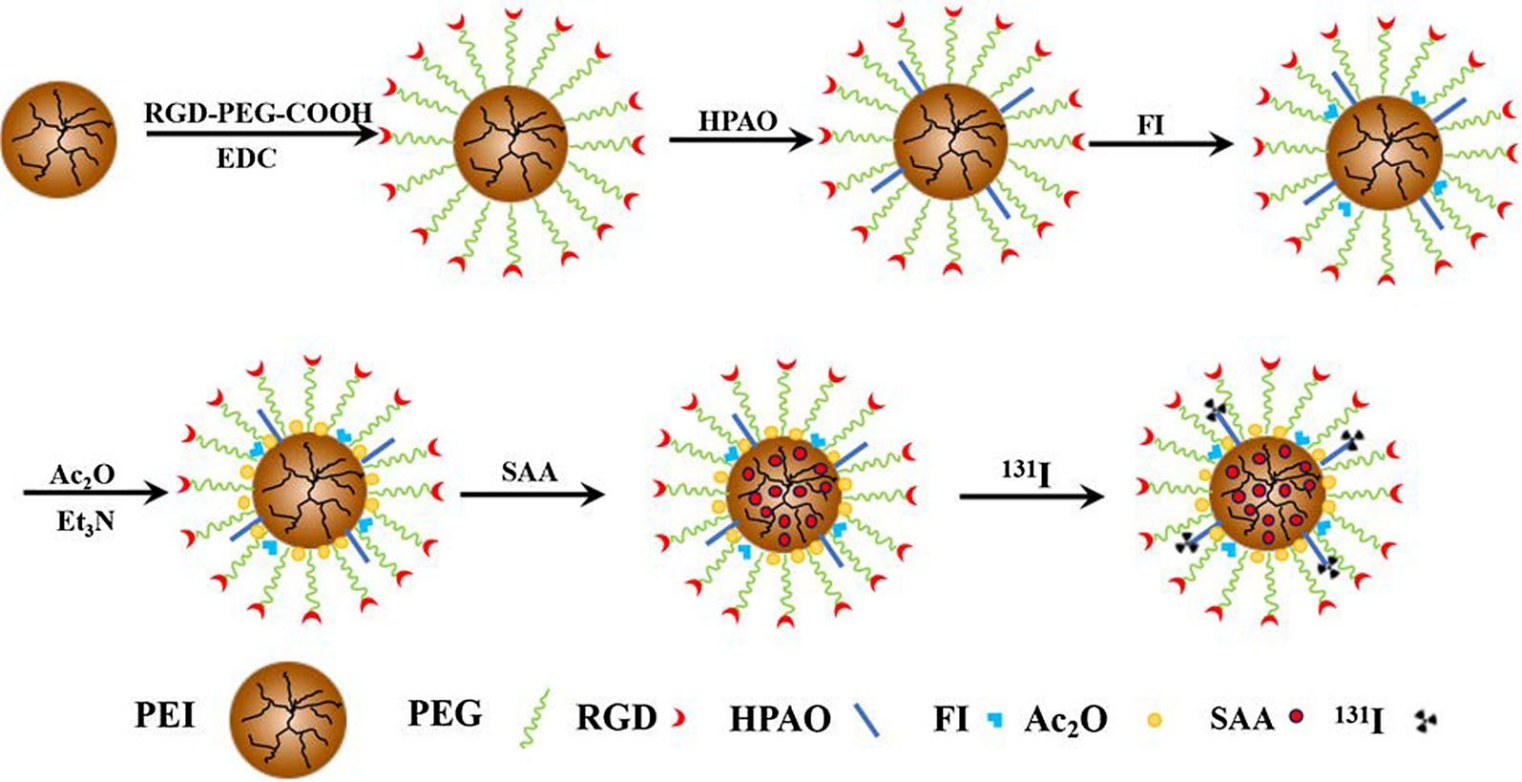
Schematic illustration of the synthesis of ^131^I-PEI.NHAc-FI-HPAO-(PEG-RGD)/SAA (^131^I-RGD-PEI/SAA)

As reported in our previous studies [32, 33, 42, 43], PEI-based nanoplatforms modified with different functional groups showed potential for the diagnosis and therapy of many diseases. Here, we designed and manufactured an RGD-modified PEI-based drug delivery nanosystem for the targeted treatment of choroidal neovascularization. Branched PEI was used as a template to link with RGD-PEG-COOH, HPAO, and FI in a stepwise manner. After acetylation of the remaining PEI surface amines using excess Ac_2_O, the formed RGD-PEI was loaded with SAA to form RGD-PEI/SAA complexes (Fig. 1). According to the structure of functional PEI, which has hydrophobic internal “cavity” formed by extending branches from a central core molecule, SAA was encapsulated into the “cavity” of RGD-PEI nanoplatform through electrostatic interaction to form RGD-PEI/SAA complexes. Through straightforward complexing process, SAA can be entrapped into multifunctional PEI for subsequent controlled release. For comparison, PEI/SAA without RGD modification was also synthesized. The final products and their corresponding intermediate products were carefully analyzed using different methods.

First, ^1^H NMR was performed to characterize the intermediate products in the synthesis process of RGD-PEI/SAA and PEI/SAA, including PEI.NH_2_-(PEG-RGD), PEI.NH_2_-HPAO-(PEG-RGD), PEI.NH_2_-FI-HPAO-(PEG-RGD), PEI.NH_2_-(*m*PEG), PEI.NH_2_-HPAO-(*m*PEG), and PEI.NH_2_-FI-HPAO-(*m*PEG) (Additional file 1: Figure S1), and the mean number of RGD, HPAO, FI, and *m*PEG moieties attached onto PEI was estimated by ^1^ H NMR integration analysis according to the protocol described in our previous studies. As shown in Additional file 1: Table S1, 3.6 RGD, 6.6 HPAO, and 4.4 FI were connected to each PEI in the PEI.NH_2_-FI-HPAO-(PEG-RGD). Similarly, the nontargeted PEI.NH_2_-FI-HPAO-(*m*PEG) nanoparticles have 13.0 *m*PEI, 6.5 HPAO, and 4.5 FI, respectively.

**Fig. 2:**
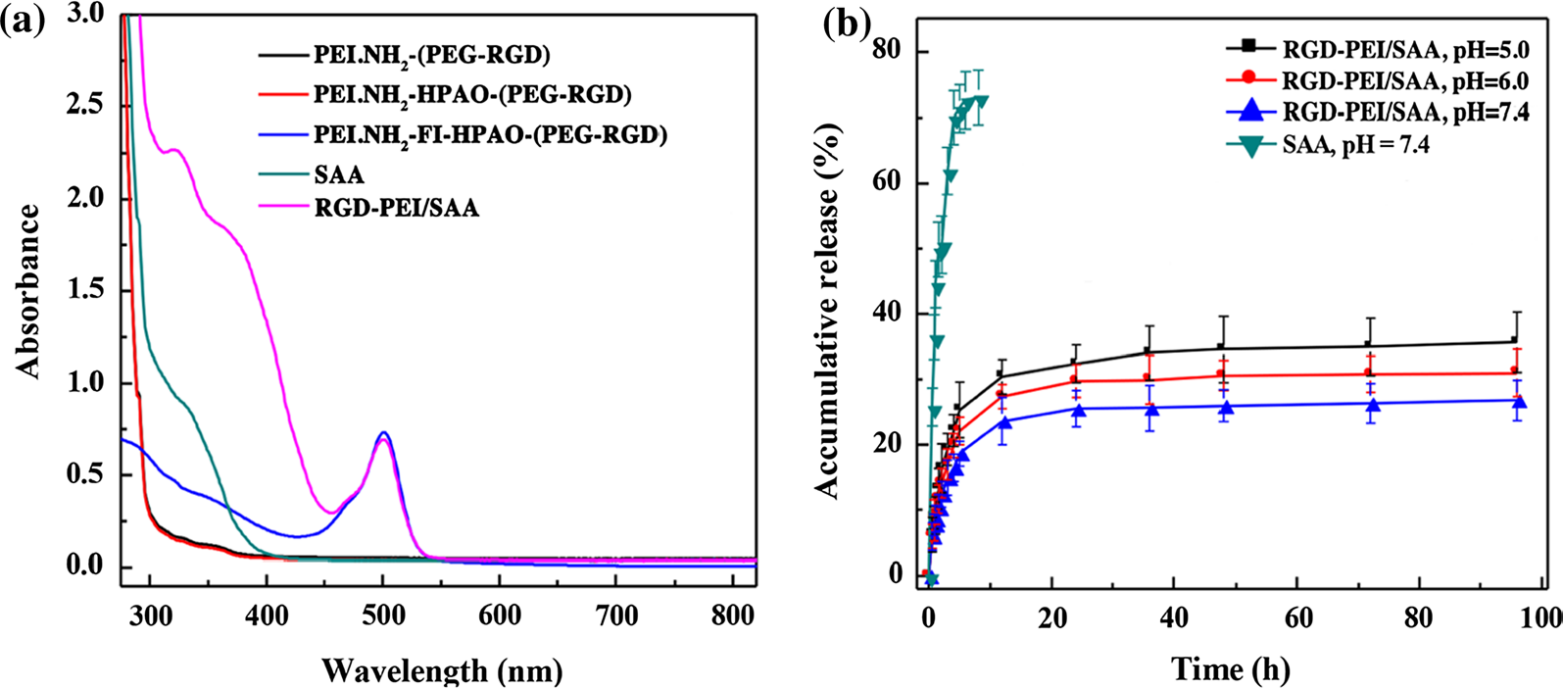
UV-Vis spectra of PEI.NH_2_-(PEG-RGD), PEI.NH_2_-HPAO-(PEG-RGD), PEI.NH_2_-FI-HPAO-(PEG-RGD), SAA, and RGD-PEI/SAA complexes (0.1 mg/mL) dispersed in water. **a** Cumulative release of SAA from RGD-PEI/SAA complexes at pH values of 5.0, 6.0, and 7.4 at 37 °C. **b** Control experiment performed by dialyzing free SAA in PBS buffer (pH = 7.4). The data are expressed as mean ± SD (*n* = 3)

Second, the remaining PEI surface amines of PEI.NH_2_-FI-HPAO-(PEG-RGD) and PEI.NH_2_-FI-HPAO-(*m*PEG) were acetylated by Ac_2_O to form RGD-PEI and *m*PEI, respectively, which were used for SAA encapsulation to synthesize RGD-PEI/SAA and *m*PEI/SAA. The synthesized RGD-PEI/SAA and *m*PEI/SAA complexes showed excellent dispersibility in different solvents such as water, cell culture media, and PBS. UV-vis spectroscopy was performed to confirm the loading of SAA and their loading efficiency. As shown in Fig. 2a, enhanced absorption at 330 nm is observed in the RGD-PEI/SAA complexes, which is in agreement with the typical absorption peak of SAA in the UV-vis spectra, while no obvious absorption is observed at this wavelength in the intermediate products without SAA loading such as PEI.NH_2_-(PEG-RGD), PEI.NH_2_-HPAO-(PEG-RGD), and PEI.NH_2_-FI-HPAO-(PEG-RGD). The amount of SAA encapsulated in RGD-PEI and *m*PEI was calculated to be 12.5 and 12.4 SAA molecules per PEI, and the SAA percentage reached 10.1 and 9.9%, respectively, suggesting a similar drug loading efficiency. In addition, the hydrodynamic size in water and zeta potential values under different pH conditions of RGD-PEI/SAA complexes were measured by dynamic light scattering. As can be seen in Additional file 1: Table S2 and Figure S2, the hydrodynamic size of RGD-PEI/SAA has relatively uniform distribution and was measured to be 316.37 ± 11.44 nm, which is slightly larger than that of PEI.NH_2_-FI-HPAO-(PEG-RGD) without SAA loading (290.53 ± 17.56 nm), suggesting the success of SAA loading. In addition, the surface potentials of RGD-PEI/SAA were similar to those of PEI.NH_2_-FI-HPAO-(PEG-RGD) under different pH conditions, reflecting that the SAA loading did not obviously change their surface potentials. Moreover, by monitoring the solution changes, the colloidal stability of RGD-PEI/SAA complexes in different solvents and pH conditions were verified, and no precipitation was observed in any of the groups during storage at 4 °C (Additional file 1: Figure S3), indicating satisfactory colloidal stability for at least 7 days.

Finally, RGD-PEI/SAA and *m*PEI/SAA could be effectively radiolabeled with ^131^I via HPAO using the chloramine T method. As shown in Additional file 1: Figures S4 and S5, The RCYs of ^131^I-RGD-PEI/SAA and ^131^I-*m*PEI/SAA were calculated to be 77.1 ± 4.4% and 72.5 ± 4.6% (*n* = 5), respectively. After purification through PD-10 desalting columns, their radiochemical purities (RCPs) were more than 99%. As to ^131^I-SAA, its RCYs was 60.1 ± 6.5% (*n* = 5), and the RCP could also be more than 99% after purification by PD MidiTrap G-10 columns. All the ^131^I-labeled compounds remained above 90% after 24 h in PBS solution at room temperature, indicating excellent stability in vitro.

### In vitro release kinetics

Frequent intravitreal injections can bring various side effects such as cataracts, endophthalmitis, retinal detachment, and hemorrhages [47]. Thus, a controlled drug delivery system that can increase the administration intervals is an unmet need in posterior neovascular ocular disease treatment. To investigate whether the RGD-PEI/SAA complex could sustainably release SAA, we examined the drug release behavior of free SAA and the RGD-PEI/SAA system under different pH conditions, including a pH of 7.4, to imitate the environment of the vitreous cavity. As shown in Fig. 2b, the free SAA released the entire dose in less than 10 h. In contrast, the RGD-PEI/SAA system showed sustained release profiles for more than 96 h. The release of RGD-PEI/SAA exhibited an initial burst in the first 5 h (approximately 25% was released) and a continued-release pattern in the following 96 h, which indicates that SAAs loaded in hyperbranched PEI tend to diffuse out on a slower time scale. These results are in line with our previous study of DOX nanocomplexes release performance [33]. The gradual release of SAA from RGD-PEI/SAA may prolong the time during which a therapeutic concentration of SAA is maintained after a single intravitreal administration, indicating its potential for the treatment of wet AMD.

### Safety study in vitro and in vivo

**Fig. 3:**
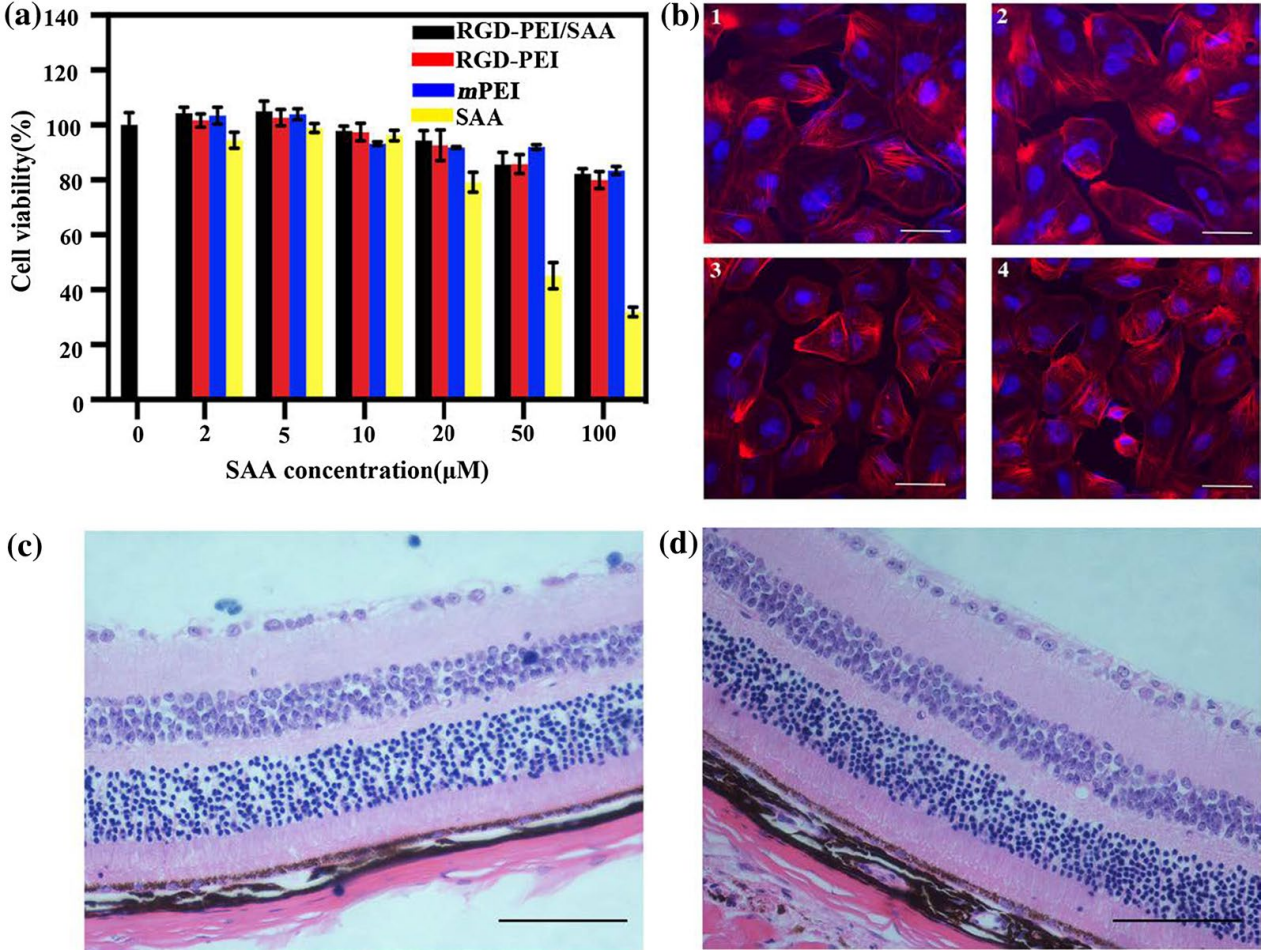
In vitro and in vivo safety assay evaluation. **a** CCK-8 assay of ARPE-19 cells treated with RGD-PEI/SAA, RGD-PEI, *m*PEI/SAA, *m*PEI, and free SAA at different SAA concentrations for 24 h. The concentrations of RGD-PEI and *m*PEI were equivalent to those of the corresponding RGD-PEI/SAA and *m*PEI/SAA complexes. **b** Fluorescence microscopy images of ARPE-19 cells co-cultured with RGD-PEI/SAA at SAA concentrations of 0 *µ*M (1), 20 *µ*M (2), 50 *µ*M (3), and 100 *µ*M (4) for 24 h (the cytoplasm and the cell nuclei were stained with phalloidin-rhodamine and DAPI, respectively). The white bar represents 20 *µ*m. Representative images of the HE stained section of retina/choroid/sclera complexes at day 7 post-intravitreal injection of PBS (**c**) and RGD-PEI/SAA (**d**) (20 *µ*M, 2 *µ*L/eye), showing no detectable injury. The scale bar is 100 *µ*m

Before performing the in vitro studies, we first tested the cytotoxicity of different forms of SAA in ARPE-19 cells by a CCK8 assay. As shown in Fig. 3a, low concentrations of SAA (≤ 20 *µ*M) did not show an obvious cytotoxic effect on ARPE-19 cells. However, higher concentrations of SAA (> 20 *µ*M) began to show an inhibitory effect on the viability of ARPE-19 cells in a dose- and time-dependent manner. The *m*PEI, RGD-PEI, and RGD-PEI/SAA did not show obvious toxicity in ARPE-19 cells at each equivalent SAA concentration. These results suggest that SAA in nanoparticle form is less toxic than the free SAA, which is likely due to the slow release of SAA from the complexes. Therefore, we chose a low concentration of SAA (20 *µ*M) for the subsequent in vivo and in vitro studies to avoid any toxicity to ARPE-19 cells.

In addition, we examined the effect on the cytoskeleton and nucleus of ARPE-19 cells after treatment with RGD-PEI/SAA at SAA concentrations of 0, 20, 50, and 100 *µ*M for 24 h. As shown in Fig. 3b, it is obvious that the cytoskeleton and nucleus of the cells treated with RGD-PEI/SAA were maintained normally at different concentrations, and no cytoskeletal injury or nucleus dysfunction is observed. These data further confirm the cytocompatibility of RGD-PEI/SAA in vitro.

Before performing in vivo experiments, we first studied the safety of RGD-PEI/SAA by single intravitreal injection in wild-type mice without any laser damage. The HE-stained section of retina/choroid/sclera complexes at day 7 post-intravitreal injection showed that, compared to the PBS group (Fig. 3c), the RGD-PEI/SAA (20 *µ*M, 2 *µ*L/eye) group (Fig. 3d) showed no evidence of inflammatory cells or loss of normal retinal structure. These toxicity study results reveal that RGD-PEI/SAA is safe both in vitro and in vivo.

### In vitro targeting specificity

**Fig. 4:**
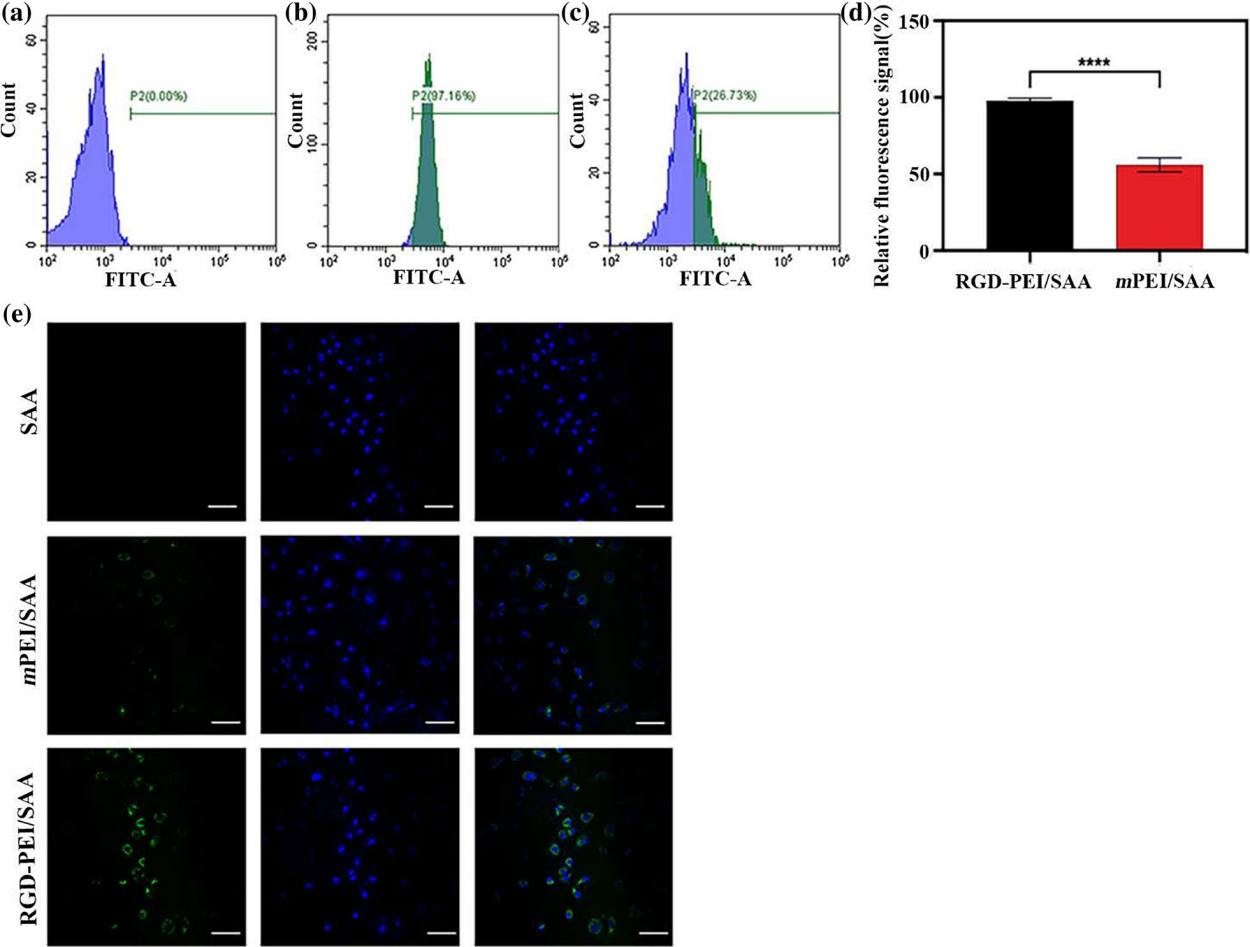
Flow cytometric analysis of ARPE-19 cells treated with **a** PBS, **b** RGD-PEI/SAA, and **c** *m*PEI/SAA complexes at an SAA concentration of 20 *µ*M for 6 h. **d** Quantitative analysis of FITC signal intensity. **e** Confocal microscopic images of ARPE-19 cells treated with PBS, *m*PEI/SAA, and RGD-PEI/SAA complexes at an SAA concentration of 20 *µ*M for 6 h

Flow cytometry and confocal laser scanning microscopy (CLSM) were used to quantitatively and qualitatively investigate the targeting specificity of RGD-PEI/SAA in vitro. As shown in Fig. 4a–d, flow cytometry analysis of ARPE-19 cells after treatment with *m*PEI/SAA and RGD-PEI/SAA at the SAA concentration of 20 *µ*M reveals that the fluorescence intensity of the RGD-PEI/SAA group is significantly higher than that of the *m*PEI/SAA group. The average numbers of FITC-positive ARPE-19 cells in mPEI/SAA and RGD-PEI/SAA were 55.93 and 97.75%, respectively. The significant increase in FITC signal after RGD modification might be due to the phagocytosis function of RPE. Under physical conditions, RPE requires ɑ_v_β_5_ integrin to recognize spent shed photoreceptor rod outer segments (POS), which is vital for retinal function [48–50]. RGD peptide, through binding to integrins including ɑ_v_β_3_ and ɑ_v_β_5_, could endow the nanoparticles with targeting ability [20, 34, 35, 39, 40]. As shown in Fig. 4e, CLSM shows that ARPE-19 incubated with RGD-PEI/SAA exhibits stronger fluorescence signals both in the cytoplasm and on the surface of the cells than the cells incubated with the equivalent amount of *m*PEI/SAA. The CLSM data are in agreement with the flow cytometry results, validating the targeting specificity of RGD-PEI/SAA in ARPE-19 cells compared with that of *m*PEI/SAA.

### In vitro anti-angiogenesis effect

**Fig. 5:**
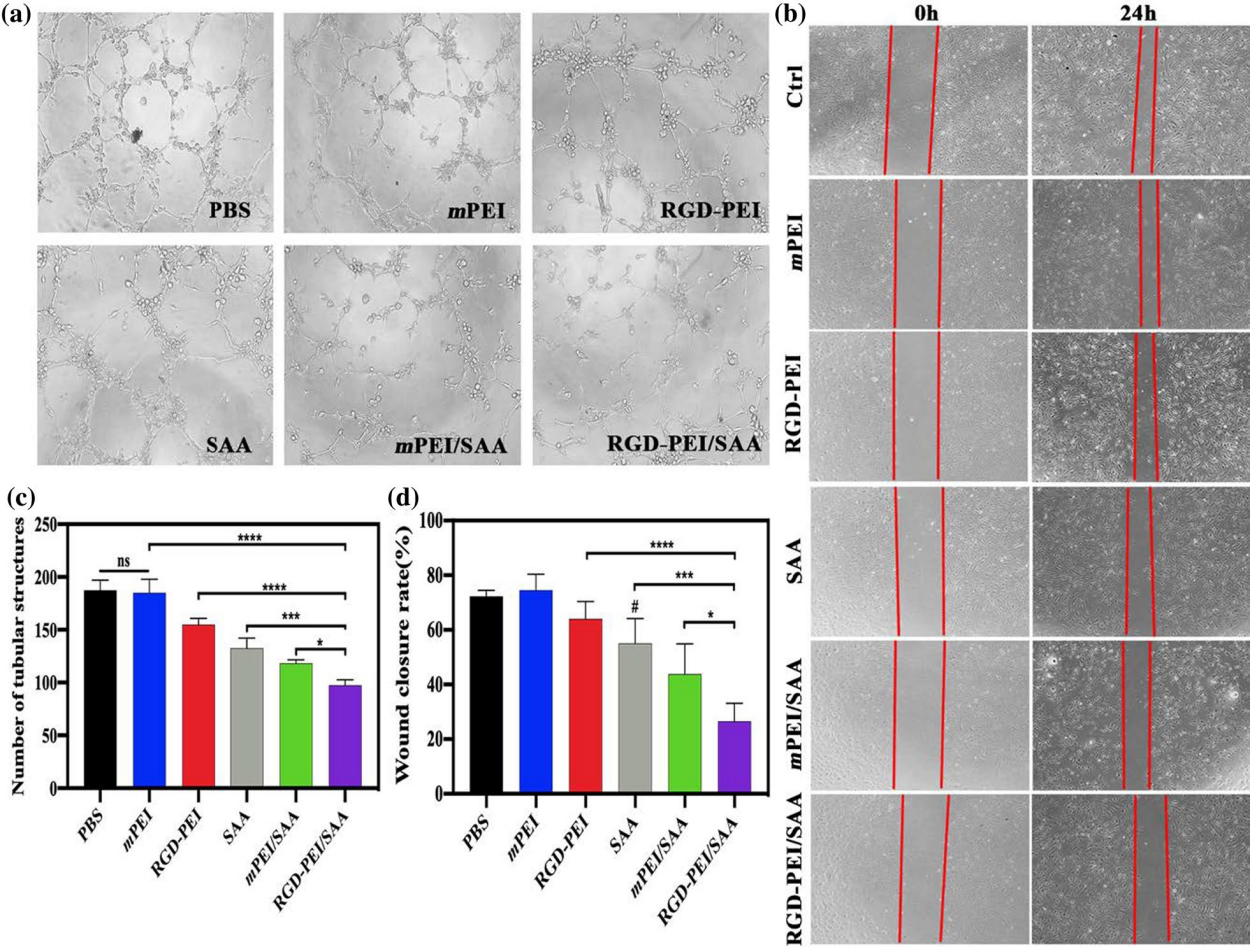
**a** Tube-like structure assay were taken 24 h after treatment (×50 magnification). **b** HUVECs incubated with PBS, *m*PEI, SAA, RGD-PEI, *m*PEI/SAA and RGD-PEI/SAA at an SAA concentration of 20 *µ*M (×50 magnification). **c** The number of tubular structures and **d** quantitative analysis of wound closure rate (* for *p* < 0.05, *** for *p* < 0.001, **** for *p* < 0.0001 versus the RGD-PEI/SAA, ^#^ for *p* < 0.05 versus mPEI/SAA )

To test the anti-angiogenesis effect of RGD-PEI/SAA in vitro, we performed a tube formation assay using HUVECs with different forms of nanoparticles at an SAA concentration of 20 *µ*M, a classic in vitro model to evaluate the effect of materials on angiogenesis, and the number of tubular structures was calculated and compared. As can be seen in Fig. 5a and c, RGD-PEI/SAA treatment shows the strongest anti-angiogenesis effect among all the groups. The groups that contained SAA and RGD, including *m*PEI/SAA, SAA, and RGD-PEI, also showed an inhibitory effect on tube formation, but they were significantly weaker than the RGD-PEI/SAA group (*p* < 0.05, *p* < 0.001, and *p* < 0.0001, respectively). However, there was no significant difference between the *m*PEI and PBS groups(p > 0.05), which indicates that the mPEI did not have an anti-angiogenesis effect in vitro, further suggesting that the anti-angiogenesis effect of RGD-PEI/SAA might be the combined effect of both RGD and SAA, but not mPEI. This result is in line with our previous study that SAA could inhibit ox-LDL-induced endothelial tube formation [27, 28], validating the anti-angiogenesis effect of SAA and the preference of RGD-PEI/SAA, which could be due to the fact that the level of integrin protein increase in angiogenic endothelial cells and the RGD-PEI/SAA could be significantly taken up by HUVECs, which is in accordance with a previous study [51].

The in vitro anti-angiogenesis effect was further validated by a scratch-wound assay on HUVEC monolayers, which is extensively used as an in vitro model to mimic the migration of endothelial cells, a crucial step in neovascular formation [44]. As shown in Fig. 5b and d, RGD-PEI/SAA (20 *µ*M)-treated cells also significantly attenuate wound closure compared to the other groups (*p* < 0.05), which is in line with the tube-like structures experiment. Although RGD-PEI, SAA, and *m*PEI/SAA treatment groups also show an inhibitory effect on HUVECs wound closure, they are obviously weaker than the RGD-PEI/SAA group, and there is no significant difference between the *m*PEI and PBS treatment groups (p > 0.05). It should also be noted that mPEI/SAA shows a preference for inhibiting wound closure compared to the free SAA(*p* < 0.05), which is likely due to its sustainable release property in vitro. Furthermore, with the modification of RGD, the anti-angiogenesis effect of mPEI was significantly enhanced (*p* < 0.05), confirming the anti-angiogenesis effect of RGD, further suggesting that the anti-angiogenesis effect of RGD-PEI/SAA is the synergistic effect of both RGD and SAA. The anti-angiogenesis effect observed in vitro laid the foundation for us to perform the following in vivo experiments.

### In vivo anti-angiogenesis efficacy

**Fig. 6:**
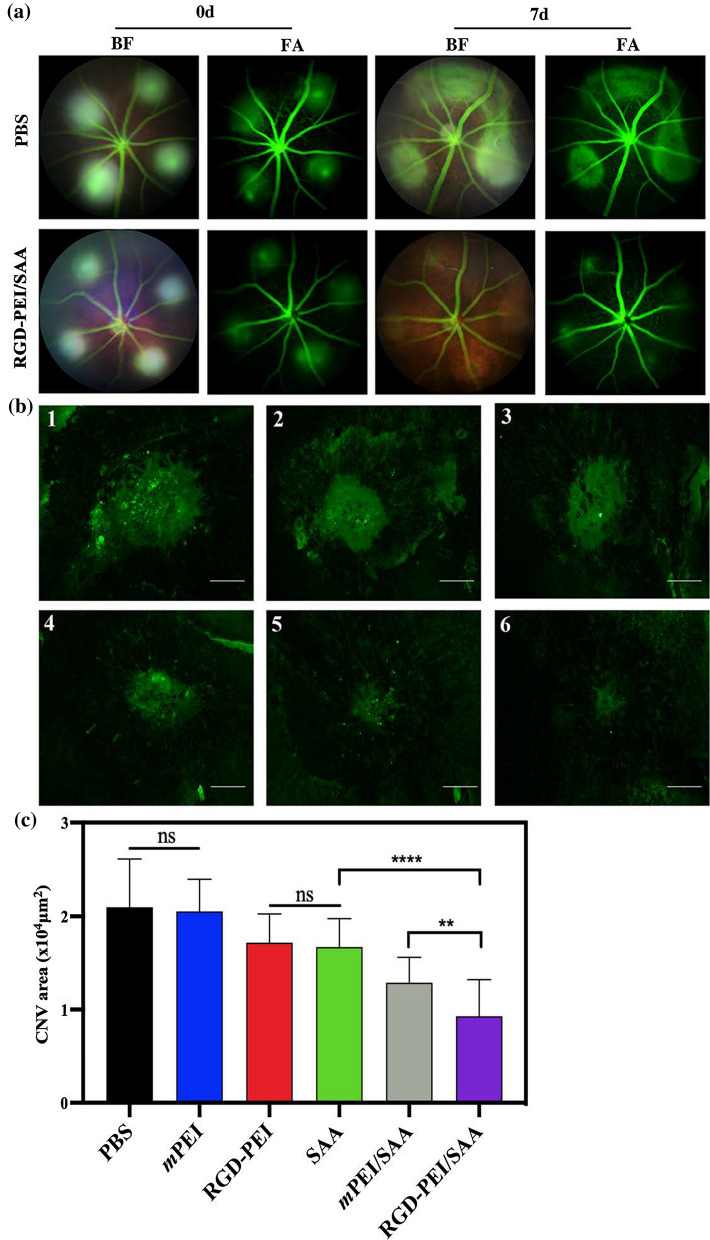
RGD-PEI/SAA reduced neovascularization in laser-induced CNV mice model. **a** Representative eye fundus photographs of BF (bright field) and FA (fluorescein angiography) obtained on Day 0 and Day 7 after laser injury with intravitreal injection of PBS or RGD-PEI/SAA (2.5 *µ*M, 2 *µ*L/eye). **b** Representative images of isolectin B4 staining laser-induced CNV RPE/choroid/sclera flat mounts after intravitreal injection of PBS (1), *m*PEI (2), RGD-PEI (3), SAA (4), *m*PEI/SAA (5) and RGD-PEI/SAA (6) at an SAA concentration of 20 *µ*M, 2 *µ*L/eye. Scale bar = 100 *µ*m. **c** Quantitative analysis of the average CNV area (mm^2^) in each group (*n* = 28). Error bar stands for the standard error of the mean (** for *p* < 0.01, **** for *p* < 0.0001)

Because of the satisfactory anti-angiogenesis effect in vitro and the sustainable release ability of RGD-PEI/SAA observed in the in vitro release kinetics study, we were interested to see whether RGD-PEI/SAA could attenuate the formation of CNV in a laser-induced mouse model, which is widely used to mimic the wet-AMD. After performing laser injury in mice, we injected SAA, RGD-PEI/SAA, *m*PEI/SAA, *m*PEI, RGD-PEI, and PBS at a concentration of SAA 20 *µ*M or the corresponding concentration of nanoparticles without SAA into the vitreous space. As the laser-induced mouse CNV model is acute, the formation of neovessels is usually between day 7 and day 14 [44]; thus we evaluated the CNV formation on day 7 after laser injury, a time point when the neovascular is thought to reach the maximum after laser. We first carried out fundus fluorescein angiography and the corresponding bright-field photography immediately after laser injury to confirm the success of laser burns and excluded those with choroidal hemorrhage or the fusion shape of more than two lesions. As shown in Fig. 6a, treatment with RGD-PEI/SAA (20 *µ*M, 2 *µ*L) showed an inhibitory effect on the formation of CNV compared to the PBS group at day 7 post laser injury in FA (fluorescein angiography) and BF (bright field) photographs. To quantitatively evaluate the CNV area in each group, the mice were sacrificed and their eyes were enucleated to prepare for the choroidal flat mounts (Fig. 6b). The CNV lesions were specifically stained with isolectin B4 (Invitrogen, Cat. No. 121,411), which is widely used as a marker of neovascular endothelial cells. The average area of the CNV lesion was calculated and compared following previous research [27, 28]. As shown in Fig. 6c, the mice treated with RGD-PEI/SAA showed a reduction of 55.78% CNV area compared to the PBS group. However, mice treated with *m*PEI/SAA, RGD-PEI, SAA, and *m*PEI showed reduction rates of 38.62%, 18.19%, 20.43%, and 2.19%, respectively, compared to the PBS group. It is obvious that RGD-PEI/SAA showed the strongest inhibitory effect on CNV formation. RGD-PEI, SAA, and *m*PEI/SAA also showed some inhibitory effect on CNV formation, but they were obviously weaker than that of RGD-PEI/SAA. There was no significant difference between the RGD-PEI and SAA groups, which indicates that both RGD and SAA show some anti-angiogenesis effect in vivo. Thus, it is reasonable to speculate that the enhanced anti-angiogenesis effect of RGD-PEI/SAA might be the combined effect of SAA and RGD.

**Fig. 7:**
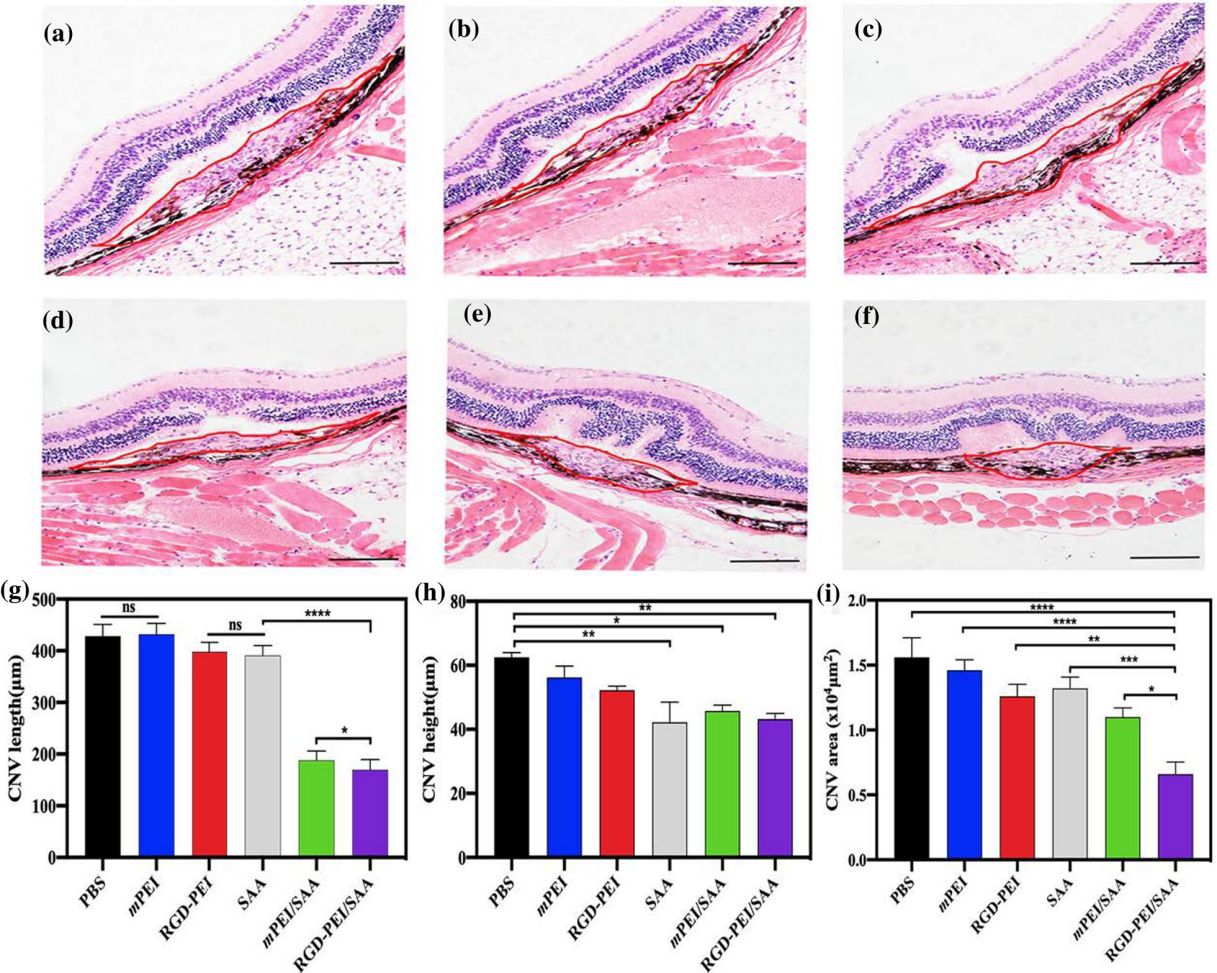
Representative images of HE staining of CNV lesions with the treatment of **a** PBS, **b** *m*PEI, **c** RGD-PEI, **d** SAA, **e** *m*PEI/SAA, and **f** RGD-PEI/SAA 7 days after photocoagulation. The red line indicates CNV areas. Scale bar = 100 *µ*m. Quantitative analysis of CNV maximum **g** length, **h** height, and **i** area of groups (∗ *p* < 0 05, **p < 0.01, ***p < 0.001, ****p < 0.0001, ns: no significance)

To confirm CNV formation in different groups, we stained and measured the CNV area using the HE assay. As can be seen in Fig. 7a–f, the Bruch’s membrane was disrupted and the newly formed vessels were in the retinal neuroepithelial layer (RNL), which disrupts the normal structure of the retina and could induce sudden bleeding in the retina or into the vitreous cavity, leading to sudden vision loss. As shown in Fig. 7 g–i, the CNV length and height, and the areas of the RGD-PEI/SAA treatment group significantly decreased compared to the other groups, which is in line with the above-mentioned choroidal flat mount staining results, confirming the inhibitory effect of RGD-PEI/SAA on angiogenesis formation in vivo. Similarly, *m*PEI/RGD, SAA, and *m*PEI/SAA treatment groups also showed some inhibitory effect on CNV formation, but they were less effective than RGD-PEI/SAA treatment. From the above data, we can see that intravitreal injection of RGD-PEI/SAA, *m*PEI/SAA, *m*PEI/RGD, and SAA showed anti-angiogenesis effects in a laser-induced CNV mouse model, among which RGD-PEI/SAA was the strongest. These results are in line with our previous studies [27, 28], confirming the anti-angiogenesis effect of SAA in a laser-induced mouse model. Furthermore, the nanoscale of SAA could increase the anti-angiogenesis effect of SAA during intravitreal administration, suggesting the advantage of nanotechnology in this study. With the modification of RGD on the surface of PEI, the anti-angiogenesis of *m*PEI/SAA was significantly increased, which might be due to not only the targeting function of RGD peptides to integrins [34, 36], but also the intrinsic anti-angiogenic activity of RGD [40, 41]. The antagonists of ɑ_v_β_3_ and ɑ_v_β_5_ are reported to block VEGFR2 phosphorylation and VEGF-stimulated activities of endothelial cells, which induce the formation of neovessels [50]. This is why we next performed the targeting CNV lesion study in mice using SPECT Imaging to further study the pharmacokinetics and biodistribution of these materials in vivo.

### RGD-PEI/SAA targeting CNV lesion in mice

**Fig. 8:**
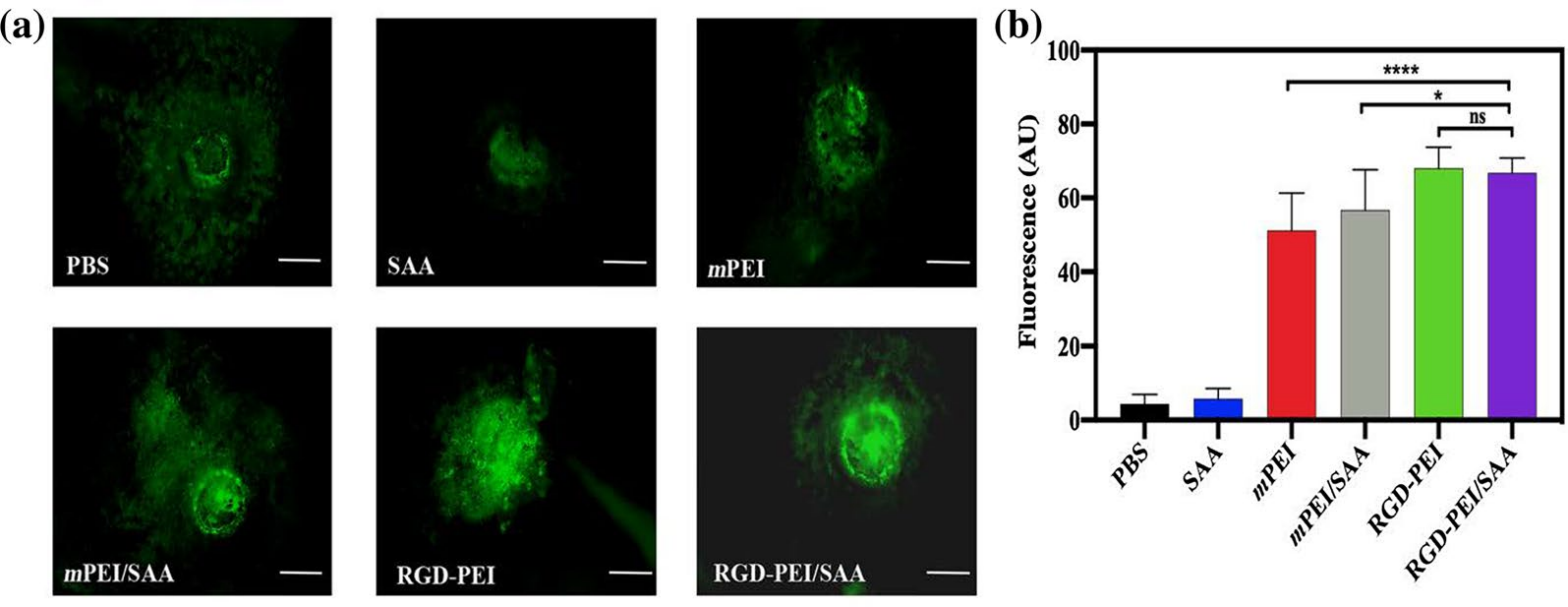
Target CNV ability of RGD-PEI/SAA and *m*PEI/SAA in mice. RGD-PEI/SAA and *m*PEI/SAA were labeled with FITC and injected (20 *µ*M, 2 *µ*L/eye) into vitreous space immediately after laser-induced CNV. **a** The RPE/choroid/sclera flat mounts were prepared 72 h after laser injury. The fluorescence in the representative photographs indicates the localization of FITC-labeled RGD-PEI/SAA and *m*PEI/SAA in the CNV lesions. The white bar represents 100 *µ*m. **b** The quantitative analysis of RPE/choroid/sclera flat mounts CNV lesions (∗*p* < 0.05, ****p < 0.0001, ns: no significance)

In order to examine the targeting efficiency of RGD-PEI/SAA in the CNV mouse model, we first labeled the nanoparticles (NPs), including RGD-PEI/SAA, RGD-PEI, mPEI/SAA, and mPEI, with FITC. Then, we injected 2 *µ*L of FITC-labeled NPs into the vitreous cavity immediately after laser injury, and the choroid flat mounts of these eyes were performed and scanned under a fluorescence microscope at 3 days post-injection. The fluorescence intensity in the laser lesion reflects the accumulation of FITC-labeled NPs. As shown in Fig. 8, the RGD-PEI/SAA and RGD-PEI groups show the highest fluorescence intensity. The *m*PEI and *m*PEI/SAA groups display significantly weaker fluorescence intensities than RGD-PEI and RGD-PEI/SAA (*p* < 0.05), suggesting that the modification of RGD could enhance the accumulation of NPs in the CNV lesions in mice, which is consistent with previous studies [46, 48, 49]. During CNV formation, integrin ɑ_v_β_3_ and ɑ_v_β_5_ are tremendously upregulated. By specifically binding to integrins in CNV lesions, RGD could facilitate the targeting accumulation of NPs in CNV sites [41, 51, 52].

### In vivo SPECT imaging in laser induced CNV mice

**Fig. 9:**
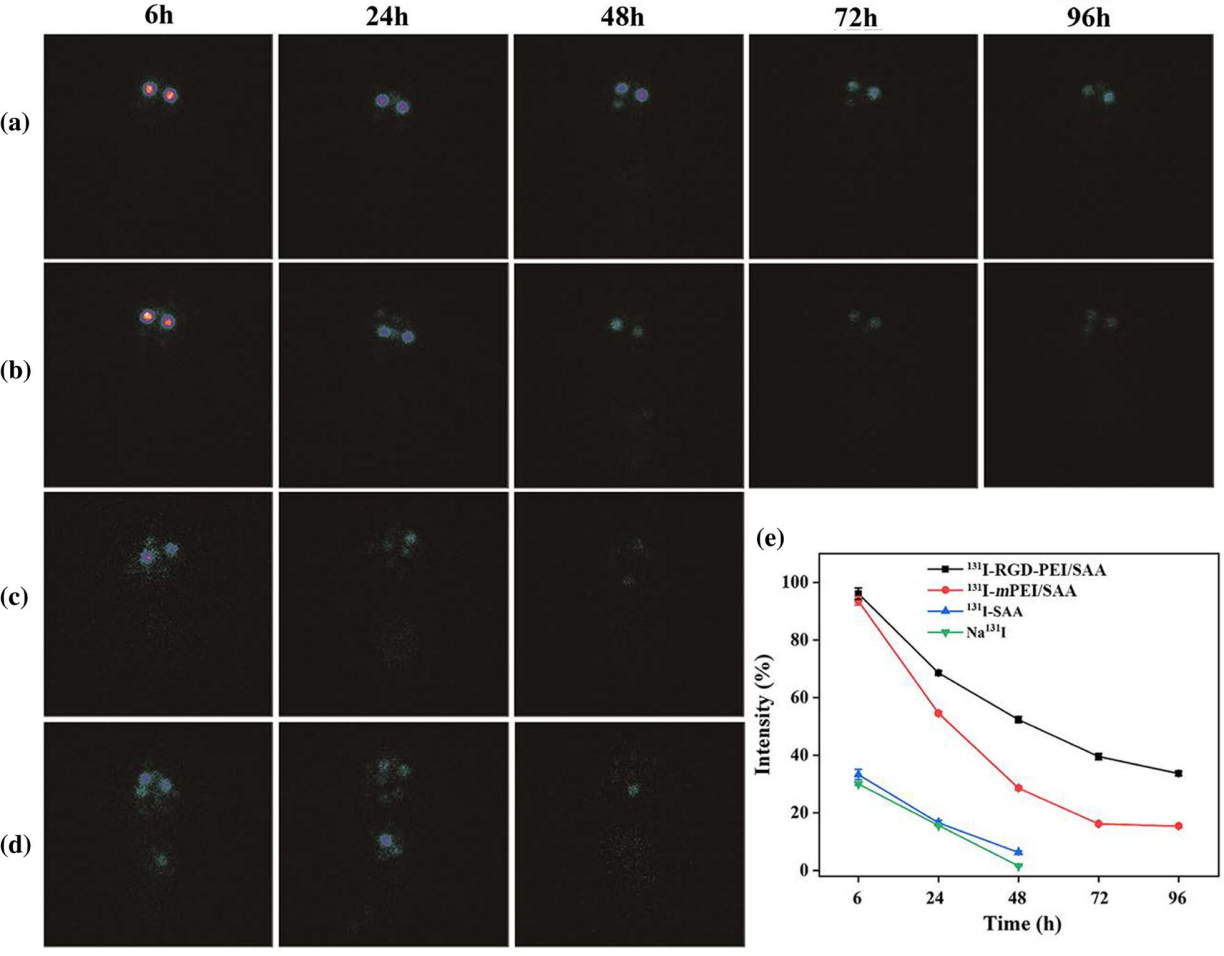
In vivo SPECT imaging of laser-induced mice after intravitreal injection of **a** ^131^I-RGD-PEI/SAA, **b** ^131^I-*m*PEI/SAA, **c** ^131^I-SAA, and **d** Na^131^I at different time points of 6, 24, 48, 72 and 96 h. **e** The relative SPECT signal intensities were quantitatively analyzed

The *m*PEI/SAA, RGD-PEI/SAA, and free SAA were radiolabeled with ^131^I for SPECT/CT imaging to further investigate their pharmacokinetics and biodistribution after intravitreal injection in the CNV mouse model. As shown in Fig. 9a–e, at 6 h after injection, the relative SPECT signal intensities of ^131^I-SAA and Na^131^I in the eyes were only 33.28 and 30.03%, while the intensities of ^131^I-RGD-PEI/SAA and^131^I-*m*PEI/SAA treatment groups were approximately 96.10 and 93.56%, respectively, revealing slow elimination of the complexes compared to free SAA. At 48 h after injection, the relative SPECT signal intensities of free SAA and free Na^131^I were 6.23 and 1.56%, respectively, while the signal intensities of ^131^I-RGD-PEI/SAA and^131^I-*m*PEI/SAA were 52.34 and 28.59%, respectively, which further confirms good retention of ^131^I-RGD-PEI/SAA in the eyes. Moreover, at 96 h after injection, the SPECT signal intensities of the ^131^I-RGD-PEI/SAA and ^131^I-*m*PEI/SAA groups were 33.65 and 15.39%, respectively, which indicates that ^131^I-RGD-PEI/SAA could stay in the vitreous space for a longer time than the corresponding form of ^131^I-*m*PEI/SAA. In addition, during the observation, the SPECT signals of ^131^I-RGD-PEI/SAA and ^131^I-*m*PEI/SAA indicated that they were accumulated mostly in the eyes and began to show signals in the thyroid gland and bladder in the ^131^I-SAA and Na^131^I groups, indicating the pharmacokinetics pathway in vivo. The results of SPECT signal intensities at different points in time further revealed the slow elimination rate of RGD-PEI/SAA in the laser-induced CNV mouse model, which is in line with the above-mentioned targeting CNV effect. The slow elimination of RGD-PEI/SAA in the vitreous cavity of laser-induced CNV mice might be beneficial for chronic posterior neovascular ocular diseases, including wet AMD, diabetic retinopathy, and retinopathy of prematurity, as it may lengthen the intravitreal injection interval period,  which requires further study both in animals and in humans. Moreover, the method used in our study to evaluate the distribution of the reagents in the CNV model was different from that used in the previous study [53] and showed several advantages. First, we could dynamically monitor the distribution of the reagents in vivo without sacrificing the mice. To our knowledge, this is the first study on the biodistribution of therapeutic reagents using SPECT imaging in the model of posterior ocular neovascular disease. Accordingly, labeling the reagents with radioisotopes, using SPECT imaging, and analyzing the corresponding SPECT signal intensity might be an optimal way to study the pharmacokinetics and biodistribution of therapeutic materials in ocular diseases, which requires further study. Secondly, as integrins were reported to be specifically expressed at CNV (not the normal retina) [41, 51], by binding to integrins in CNV lesions, RGD-modified NPs labeling with radioisotopes and detection with SPECT imaging might be a way to outline the area or volume of CNV in vivo, which could be a useful application for monitoring the development of CNV in patients, and requires more research both in animals and in humans.

## Conclusions

In this study, we designed and constructed a multifunctional PEI encapsulating SAA for targeted anti-angiogenesis therapy. Our results revealed that the RGD-PEI/SAA system could release SAA in a sustainable manner. With decoration of the RGD peptide, the RGD-PEI/SAA system could target integrin-expressing ARPE-19 cells in vitro and CNV lesions in vivo. With good safety results both in vitro and in vivo, the RGD-PEI/SAA NPs might be a promising targeted anti-angiogenesis therapy for chronic neovascular posterior ocular diseases. In addition, this nanoparticle system could be a good vehicle to load other antiangiogenic drug which requires further research.

## Supplementary Information

**Additional file 1.** Part of experimental details: Materials; Characterization techniques; In vitro cytotoxicity assay and cytoskeleton observation; Flow cytometry assay of the specific cellular uptake; Confocal laser scanning microscopy (CLSM); Statistical analysis. **Figure S1.** ^1^H NMR spectra of intermediate products in the synthesis process of RGD-PEI/SAA and PEI/SAA. **Table S1.** The drug loading efficiency of SAA in mPEI/SAA complexes and RGD-PEI/SAA complexes. **Table S2.** The hydrodynamic size of PEI.NH_2_-FI-HPAO-(PEG-RGD) and RGD-PEI/SAA complexes dispersed in water. **Figure S2.** The hydrodynamic size distribution of PEI.NH_2_-FI-HPAO-(PEG-RGD) and RGD-PEI/SAA complexes dispersed in water. **Table S3.** Zeta potential values of PEI.NH_2_-FI-HPAO-(PEG-RGD) and RGD-PEI/SAA complexes under different pH conditions. **Figure S3.** Photograph of the RGD-PEI/SAA complexes dispersed in different solvents and pH conditions at the concentration of 0.5 mg/mL. **Figure S4.** Radiochemical purity of ^131^I-RGD-PEI/SAA and ^131^I-mPEI/SAA at different time points tested by instant thin-layer chromatography (ITLC). **Figure S5.** The radiochemical purities (RCPs) of ^131^I-RGD-PEI/SAA and ^131^I-mPEI/SAA recorded at 37 °C for different time.

## Data Availability

All data generated or analysed during this study are included in this published article.
