# Supplementary material for: RGD-modified multifunctional nanoparticles encapsulating salvianolic acid A for targeted treatment of choroidal neovascularization
Source: J Nanobiotechnology. 2021 Jul 2;19:196. doi: 10.1186/s12951-021-00939-9 (PMC8254347; doi:10.1186/s12951-021-00939-9)
Supplement: Supplementary file 1 — Additional file 1. Part of experimental details: Materials; Characterization techniques; In vitro cytotoxicity assay and cytoskeleton observation; Flow cytometry assay of the specific cellular uptake; Confocal laser scanning microscopy (CLSM); Statistical analysis. Figure S1. 1H NMR spectra of intermediate products in the synthesis process of RGD-PEI/SAA and PEI/SAA. Table S1. The drug loading efficiency of SAA in mPEI/SAA complexes and RGD-PEI/SAA complexes. Table S2. The hydrodynamic size of PEI.NH2-FI-HPAO-(PEG-RGD) and RGD-PEI/SAA complexes dispersed in water. Figure S2. The hydrodynamic size distribution of PEI.NH2-FI-HPAO-(PEG-RGD) and RGD-PEI/SAA complexes dispersed in water. Table S3. Zeta potential values of PEI.NH2-FI-HPAO-(PEG-RGD) and RGD-PEI/SAA complexes under different pH conditions. Figure S3. Photograph of the RGD-PEI/SAA complexes dispersed in different solvents and pH conditions at the concentration of 0.5 mg/mL. Figure S4. Radiochemical purity of 131I-RGD-PEI/SAA and 131I-mPEI/SAA at different time points tested by instant thin-layer chromatography (ITLC). Figure S5. The radiochemical purities (RCPs) of 131I-RGD-PEI/SAA and 131I-mPEI/SAA recorded at 37 °C for different time. [file 12951_2021_939_MOESM1_ESM.docx]

Supporting Information

RGD-Modified Multifunctional Nanoparticles Entrapping Salvianolic Acid A for Targeted Treatment of Choroidal Neovascularization

**Junxiu Zhang**^1‡^**, Jingyi Zhu**^2‡^**, Lingzhou Zhao**^3‡^**, Ke Mao**^4^**, Qing Gu**^1^**, Jinhua Zhao**^3*^ **and Xingwei Wu**^1*^

^1^Departmentof Ophthalmology, Shanghai General Hospital, Shanghai Jiao Tong University School of Medicine, Shanghai Key Laboratory of Ocular Fundus Diseases, Shanghai Engineering Center for Visual Science and Photomedicine, Shanghai, 200080, People’s Republic of China

^2^School of Pharmaceutical Sciences, Nanjing Tech University, Nanjing 211816, People’s Republic of China

^3^Department of Nuclear Medicine, Shanghai General Hospital, Shanghai Jiao Tong University School of Medicine, Shanghai 200080, People’s Republic of China

^4^Department of Ophthalmology, Renji Hospital, School of Medicine, Shanghai Jiao Tong University, Shanghai 200127, People’s Republic of China

*Corresponding Authors. Email address: wxweye@sina.com (X.W.); zhaojinhua1963@126.com (J.Z.).

**‡**These authors equally contributed to this work.

Part of experimental details:

**Materials.** Branched PEI (Mw = 25 000), acetic anhydride (Ac_2_O),dimethyl sulfoxide (DMSO), PEG monomethyl ether with one end of carboxyl group (*m*PEG-COOH, Mw = 2 000), dual functional PEG with one end of the carboxyl group and the other end of an amine group (NH2-PEG-COOH, Mw = 2 000), RGD peptide, HPAO were purchased from Sigma-Aldrich (St. Louis, MO).Na^131^I was supplied by Shanghai GMS Pharmaceuticals (Shanghai, China). SAA was purchased from Nanjing Guangrun Biochemical Company (Nanjing, China). The human RPE cell line (ARPE-19) and human umbilical vein endothelial cells (HUVECs) were from the Institute of Biochemistry and Cell Biology (the Chinese Academy of Sciences, Shanghai, China). Phosphate buffered saline (PBS), fetal bovine serum (FBS), RPMI 1640 medium, penicillin, and streptomycin were procured from Shanghai Dobio CO., Ltd. (Shanghai, China). Matrigel (356234) were obtained from Corning (Corning, NY). Phalloidin and 4',6-diamidino-2-Phenylindole (DAPI) were purchased from Servicebio (Wuhan, China). Cell counting kit-8 (CCK-8) was procured from Biosharp (Shanghai, China). All other chemicals and solvents were supplied by 7sea Biotech. Co., Ltd. (Shanghai, China).

**Characterization techniques**. ^1^H NMR spectra of samples were dissolved into D_2_O before measured on a Bruker AV400 nuclear magnetic resonance spectrometer (Bruker AXS Advanced X-ray Solutions GmbH, Karlsruhe, Germany). UV-vis spectra were performed using a Lambda 25 UV-vis spectrophotometer (PerkinElmer, Inc., Waltham, MA, USA). Hydrodynamic sizes and zeta potential were carried out using a Malvern Zetasizer Nano ZS model ZEN 3600 (Malvern Panalytical Ltd., Malvern, UK) with a standard 633 nm laser. The size and morphology of atomic force microscopy (AFM, Veeco DI-NanoScopy IV, Santa Barbara, CA) by tapping mode using a rotated monolithic silicon probe. The parameters were set as following: Imaging Mode (AC mode), scan lines (256), scan point (256), scan rate (1.00Hz), and scan size (3.00 μm).A GE Infinia SPECT scanner equipped with a Xeleris workstation and low-energy general-purpose collimators (GE Healthcare) was used to obtain SPECT imaging.

**In vitro cytotoxicity assay and cytoskeleton observation.** Cell counting kit (CCK)-8 assay was used to examine the cytotoxicity effects of RGD-PEI/SAA, *m*PEI-SAA on the ARPE-19 cells. ARPE-19 cells were cultured in 96-well plates at a density of 4 × 10^3^ cells/well. After starved for 24 h, the culture media were replaced with 100 μL fresh media containing various concentrations of SAA (0, 2, 5, 10, 20, 50, 100 μM) or the corresponding concentrations of RGD-PEI/SAA, *m*PEI/SAA containing the same concentrations of SAA for another 24 h. Then 10 μL of CCK-8 assay was added in each well, and the cells were continued incubating for 4 h. The absorbance was read at 450 nm using a multimode microplate reader (Molecular Devices Spectramax M5). The cell viability was calculated following the protocol described before. Each experiment was performed three times.

Similarly, ARPE-19 cells (5 ×10^4^ cells/well) were seeded on coverslips with 1mL of fresh DMEM for each well for 24 h. Then the cells were incubated with a fresh medium containing RGD-PEI/SAA at SAA 0，20，50，100 μM for 24 h, after rinsed with PBS, the cells were fixed with 4.0% paraformaldehyde for 30 min at room temperature. After washed with PBS for another 3 times, the cells were stained with phalloidin-rhodamine. Finally, the cells were washed using PBS for 3 times, stained with DAPI for 10 min, and washed subsequently before light microscopic imaging.

**Flow cytometry assay of the specific cellular uptake.** Flow cytometry was performed to evaluate the targeting specificity of RGD-PEI/SAA for ARPE-19 cells. Typically, after starving for 24 h, ARPE-19 cells were incubated RGD-PEI/SAA, *m*PEI/SAA , PBS at 20 μM SAA for 12 h. Then the cells were washed with PBS, detached with 0.25% Trypsin-EDTA solution, suspended using trypsin neutralizing solution and were analyzed by BD LSR Fortessa cell analyzer (BD Bioscience, NJ, USA).

**Confocal laser scanning microscopy (CLSM)** ARPE-19 cells were seeded at 2 × 10^5^ cells per well in a 33-mm confocal dish. After starving for overnight, each well was treated with RGD-PEI/SAA, *m*PEI/SAA, PBS at 20 μM SAA for 12 h. Then the cells were washed with cold PBS, fixed with 4% paraformaldehyde and the cell nuclei were stained with Dapi. Confocal microscopy pictures were captured using a Carl Zeiss LSM 700 laser scanning confocal microscope (Jena, Germany).

**Statistical analysis.**  One-way analysis of variance (ANOVA) statistical analysis was used to assess the significance of the experimental data. Significance level was set at 0.05, and the results were indicated as *p < 0.05, **p < 0.01, ***p < 0.001, and ****p < 0.001, respectively.


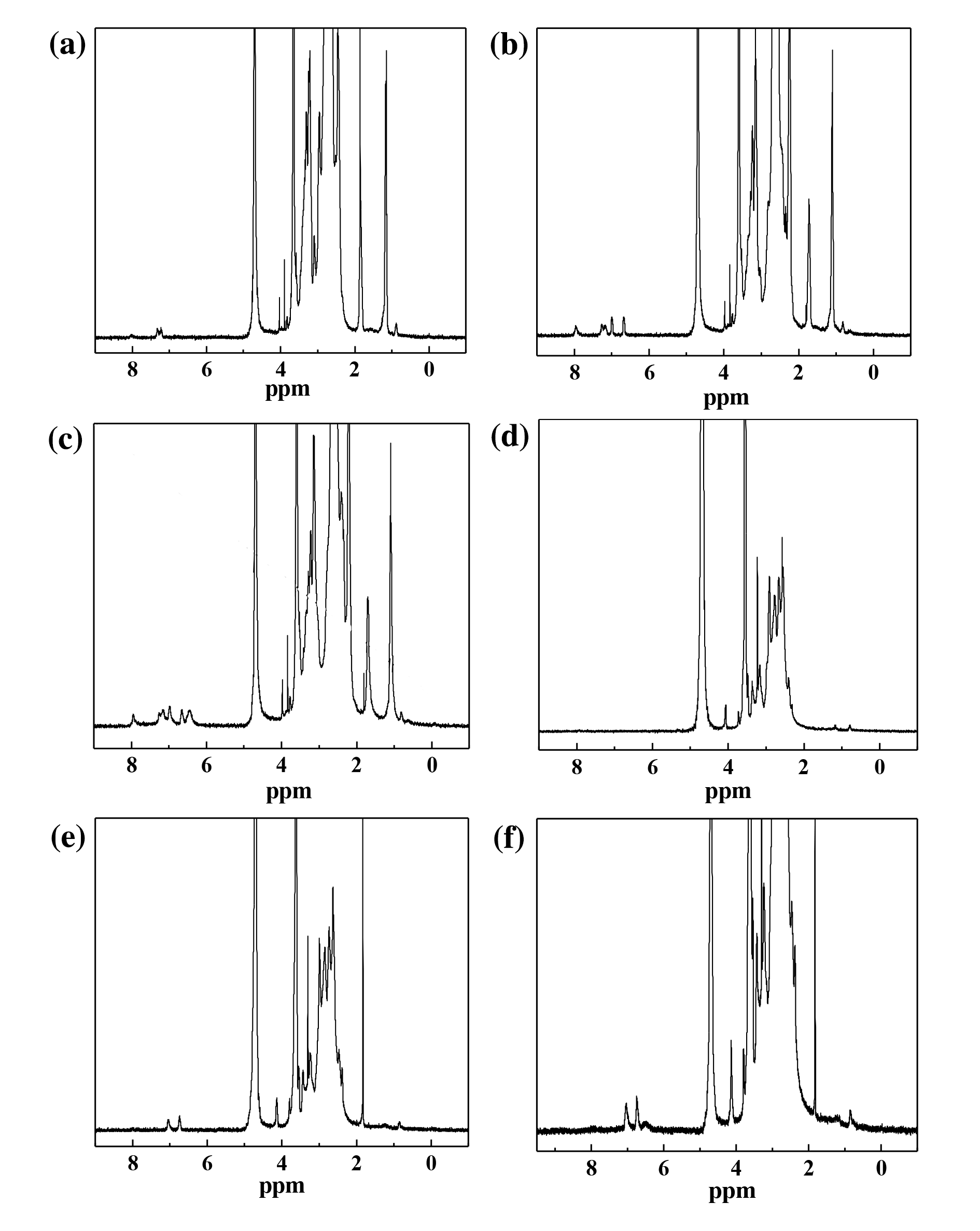


**Figure S1**. ^1^H NMR spectra of PEI.NH_2_-(PEG-RGD) (a) and PEI.NH_2_-HPAO-(PEG-RGD) (b) PEI.NH_2_-FI-HPAO-(PEG-RGD) (c) PEI.NH_2_-(*m*PEG) (d) PEI.NH_2_-HPAO-(*m*PEG) (e), and PEI.NH_2_-FI-HPAO-(*m*PEG) (f). 3.6 RGD, 6.6 HPAO, and 4.4 FI were connected to each PEI in the PEI.NH_2_-FI-HPAO-(PEG-RGD). The nontargeted PEI.NH_2_-FI-HPAO-(*m*PEG) nanoparticles have 13.0 *m*PEG, 6.5 HPAO, and 4.5 FI moieties.

**Table S1**. The drug loading efficiency of SAA in mPEI/SAA complexes and RGD-PEI/SAA complexes

| **Materials** | **SAA percentage (wt.%)** | **Number of complexed SAA per PEI** |
| --- | --- | --- |
| **mPEI/SAA** | **10.10%** | **12.37** |
| **RGD-PEI/SAA** | **9.87%** | **12.52** |

**Table S2**. The hydrodynamic size of PEI.NH_2_-FI-HPAO-(PEG-RGD) and RGD-PEI/SAA complexes dispersed in water.

| **Materials** | **Hydrodynamic size (nm)** | **PDI** |
| --- | --- | --- |
| **PEI.NH2-FI-HPAO-(PEG-RGD)** | **290.53±17.56** | **0.42±0.06** |
| **RGD-PEI/SAA** | **316.37±11.44** | **0.47±0.11** |


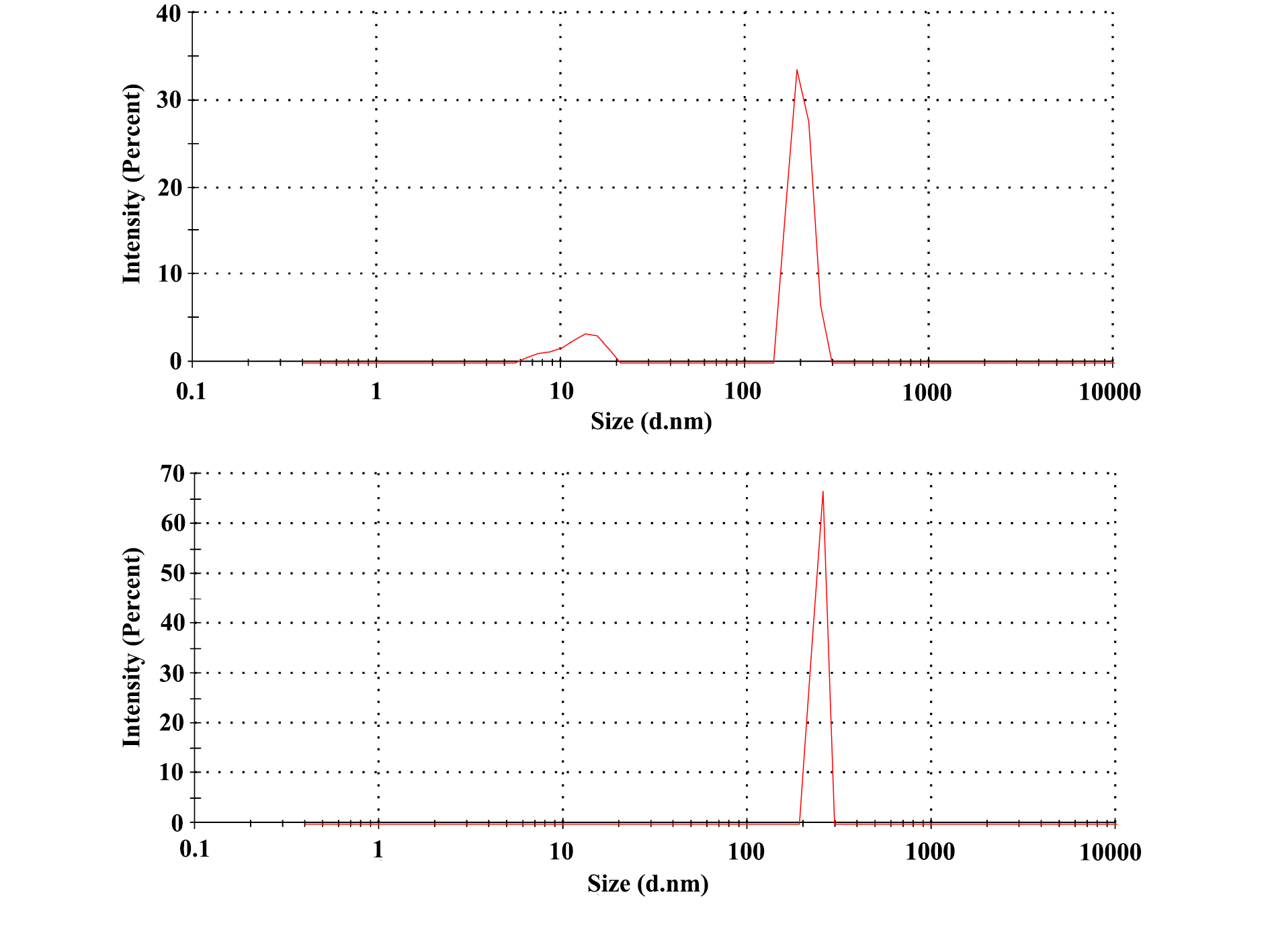


**Figure S2**. The hydrodynamic size distribution of PEI.NH_2_-FI-HPAO-(PEG-RGD) (a) and RGD-PEI/SAA complexes (b) dispersed in water.

**Table S3**. Zeta potential values of PEI.NH_2_-FI-HPAO-(PEG-RGD) and RGD-PEI/SAA complexes under different pH conditions.

| **Materials** | **Zeta potential (mV)** | | | |
| --- | --- | --- | --- | --- |
|  | **pH=5.0** | **pH=6.0** | **pH=7.4** | **pH=10.0** |
| **PEI.NH2-FI-HPAO-(PEG-RGD)** | **0.42±0.06** | **0.42±0.06** | **0.42±0.06** | **0.42±0.06** |
| **RGD-PEI/SAA** | **0.42±0.06** | **0.42±0.06** | **0.42±0.06** | **0.42±0.06** |


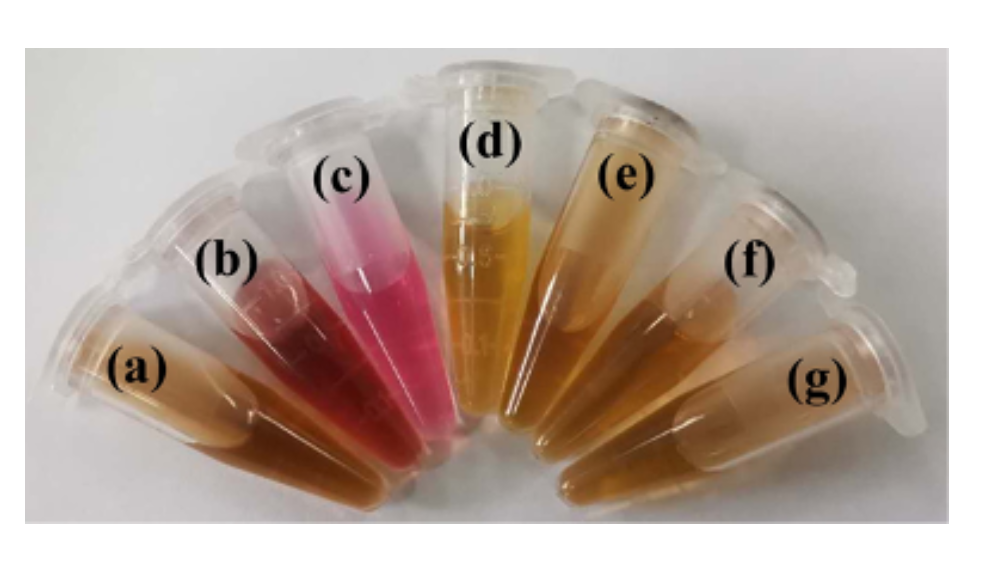


**Figure S3**. Photograph of the RGD-PEI/SAA complexes dispersed in water (a), cell culture medium (b), and PBS with pH 5.0 (d), 6.0 (e), 7.4 (f) and 10.0 (g) at the concentration of 0.5 mg/mL, respectively. (c) shows the blank cell culture medium.


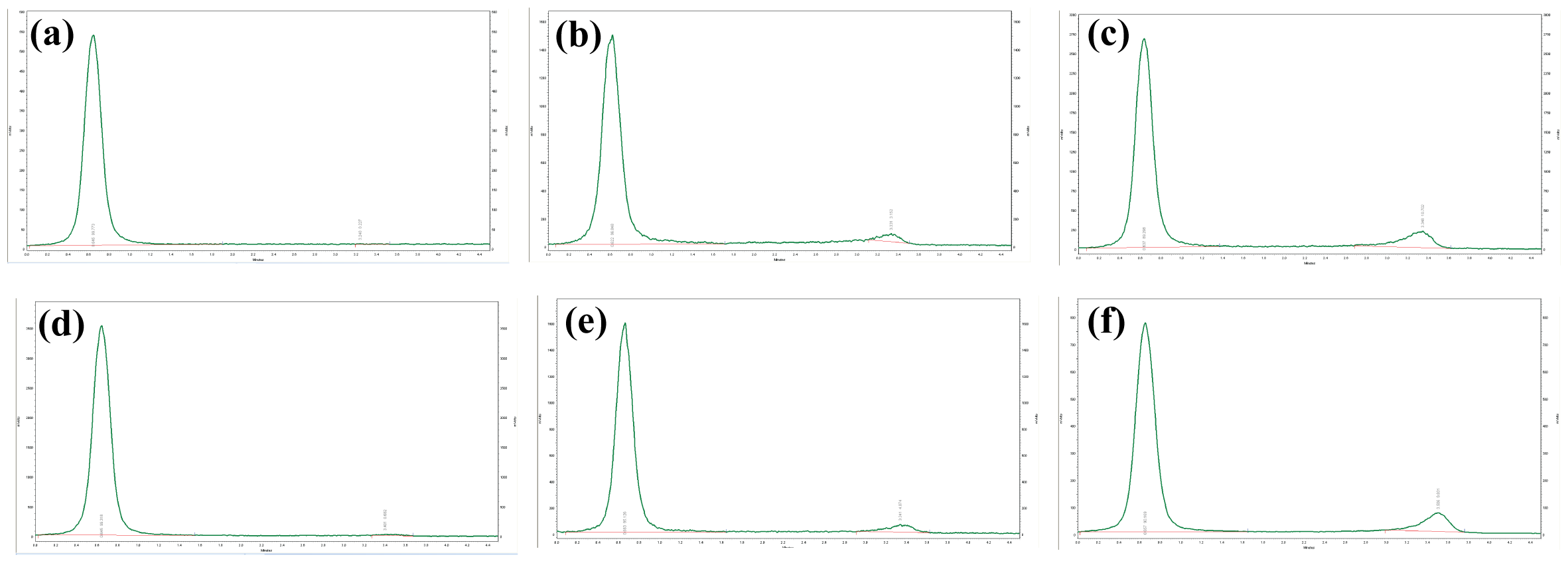


**Figure S4**. Radiochemical purity of ^131^I-RGD-PEI/SAA at (a) 1 h, (b) 6 h, (c) 24 h, and ^131^I-*m*PEI/SAA at (d) 1 h, (e) 6 h, (f) 24 h, which was tested by instant thin-layer chromatography (ITLC).


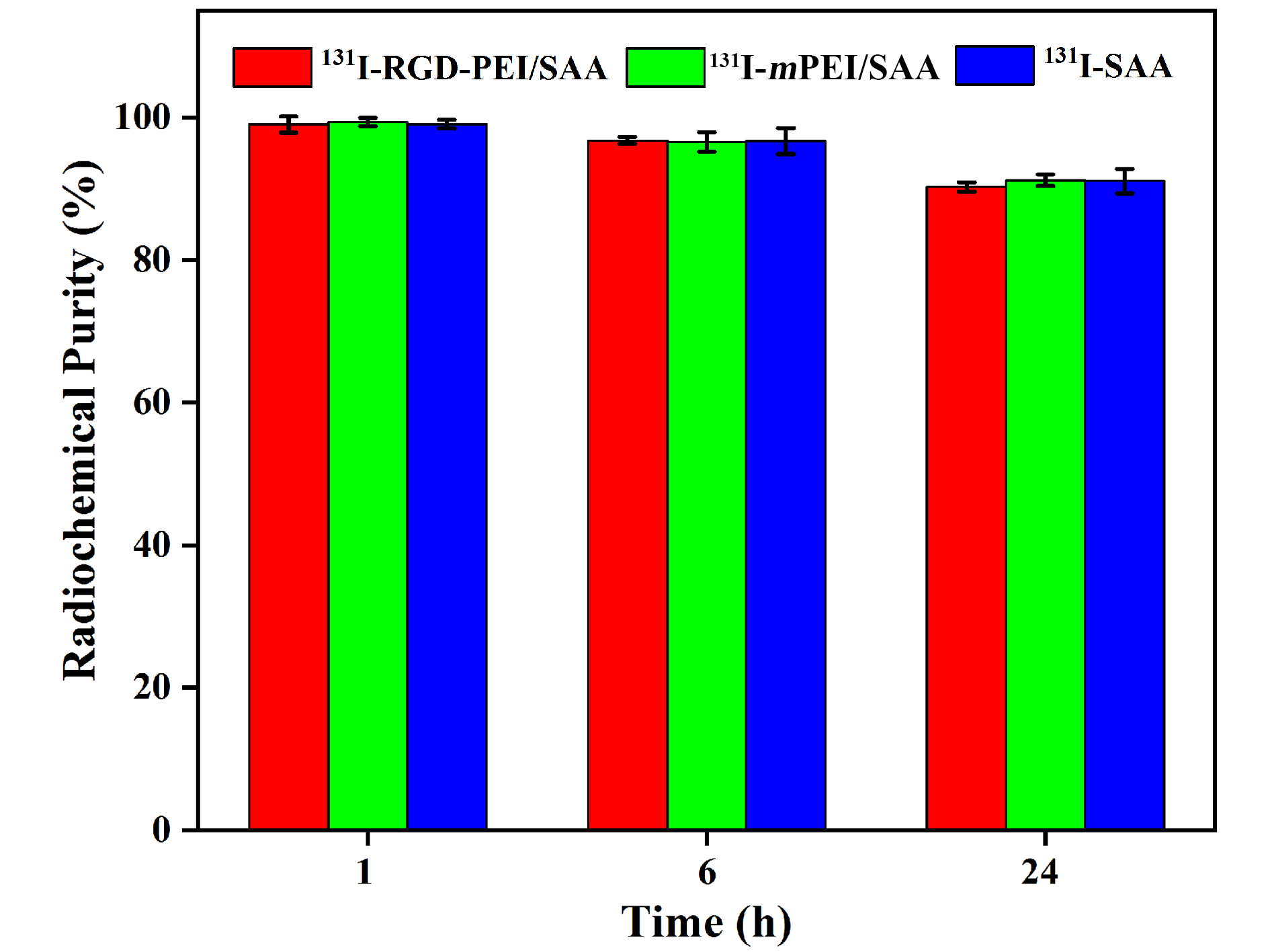


**Figure S5.** The radiochemical purities (RCPs) of ^131^I-RGD-PEI/SAA and ^131^I-*m*PEI/SAA were recorded at 37 °C for 1, 6 and 24 h, respectively.
